# Hapten sensitization to vaginal mucosa induces less recruitment of dendritic cells accompanying TGF‐β‐expressing CD206^+^ cells compared with skin

**DOI:** 10.1002/iid3.605

**Published:** 2022-03-10

**Authors:** Kanako Nakayama, Taku Nishijo, Masaaki Miyazawa, Tetsuro Watabe, Miyuki Azuma, Hitoshi Sakaguchi

**Affiliations:** ^1^ Safety Science Research Laboratories, Kao Corporation Haga Tochigi Japan; ^2^ Department of Biochemistry Graduate School of Medical and Dental Sciences, Tokyo Medical and Dental University Bunkyo‐ku Tokyo Japan; ^3^ Department of Molecular Immunology Graduate School of Medical and Dental Sciences, Tokyo Medical and Dental University Bunkyo‐ku Tokyo Japan

**Keywords:** contact hypersensitivity, dendritic cells, haptens, skin, vaginal mucosa

## Abstract

**Introduction:**

Contact hypersensitivity (CHS), a type of delayed‐type hypersensitivity, is induced by hapten exposure to the skin and mucosa. We previously reported that, in a murine model of CHS, the vaginal mucosa (VM) sensitization showed lower T‐cell responses as compared with the abdominal skin sensitization. To investigate mechanisms of impaired CHS by the VM sensitization, we compared migration of hapten‐captured dendritic cells (DCs) in the draining lymph nodes (dLNs) and recruitment of DCs at the sensitized local sites.

**Methods:**

Fluorescein isothiocyanate (FITC) or 2,4‑dinitrofluorobenzene (DNFB) was used as hapten, and migration of FITC^+^ DCs in the dLNs and local recruitment of MHC class II^+^ and CD11c^+^ cells were compared between abdominal skin and VM sensitization by flow cytometric analyses and immunohistochemistry. Expression of tumor growth factor (TGF)‐β at mRNA and protein levels, and local recruitment of CD206^+^ cells were examined after VM sensitization.

**Results:**

VM sensitization showed less numbers of FITC^+^MHC class II^high^CD11c^+^ migratory DCs in the dLNs at 6 and 24 h, as compared with skin sensitization. Both skin and VM sensitization induced the recruitment of dermal/submucosal DCs at 6 h, but the number of submucosal DCs in the VM was significantly decreased at 24 h. VM showed persistently higher mRNA levels of TGF‐β2/β3 expression than those of the skin before and after sensitization. In the VM sensitization, increment of CD206^+^MHC class II^+^ cells was observed especially at the deep lamina propria at 24 h. Most of CD206^+^ cells were also positive for the binding to Fc chimeric TGF‐β receptor that interacts with all TGF‐β isoforms, suggesting TGF‐β expression.

**Conclusion:**

DC migration to dLNs and localization of DCs at the sensitized sites are limited in the VM sensitization. Our results suggest that the existence of TGF‐β‐expressing CD206^+^ cells may contribute less sensitization ability and CHS responses in the VM.

## Graphical abstract

To investigate mechanisms of vaginal mucosa (VM) sensitization in contact hypersensitivity (CHS), we examined migration of hapten‐captured dendritic cells (DCs) in the draining lymph nodes (dLNs) and recruitment of DCs at the sensitized local sites in VM sensitization as compared with skin sensitization. We observed that DC migration to dLNs and localization of DCs at the sensitized sites were limited in the VM sensitization with high TGF‐β expression levels, increment of CD206^+^ cells, and TGF‐β expression in CD206^+^ cells in the VM. These results suggested that existence of TGF‐β‐expressing CD206^+^ cells may contribute less sensitization ability and CHS responses in the VM.

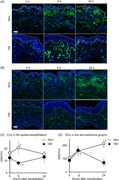

## 1 INTRODUCTION

Contact allergy, a delayed‐type hypersensitivity, can be caused by skin and mucosal exposure to low‐molecular‐weight sensitizing chemicals called haptens. The mechanism of skin contact allergy, which consists of sensitization and elicitation phases, has been actively investigated using a murine contact hypersensitivity (CHS) model.^1^, ^2^ In the sensitization phase of CHS and upon hapten application, dendritic cells (DCs) efficiently acquire antigen (hapten‐carrier conjugate) in the skin. Antigen‐captured DCs migrate to draining lymph nodes (dLNs), while DCs are newly recruited at the sensitization site in the peripheral tissue from the blood. Migratory DCs present the antigen to naïve T cells and induce T‐cell proliferation and differentiation in dLNs. In the subsequent elicitation phase, antigen‐specific T cells are infiltrated by re‐exposure to haptens and elicit skin inflammation. DCs have a critical role in the development of sensitization in contact allergies and are mainly categorized into two subsets based on their anatomical location: Langerhans cells (LCs) in the epidermis and dermal DCs in the dermis.^1^, ^2^, ^3^, ^4^ LCs and dermal DCs play a role in inducing T‐cell differentiation in CHS, while LCs play a regulatory role in the sensitization phase of CHS.^1^, ^3^, ^4^

Mucosae, such as the oral and vaginal cavities, are also inductive sites of contact allergy caused by hapten exposure.^5^, ^6^, ^7^ Compared with skin, there have been far fewer studies on contact allergy in the mucosae, especially in the vaginal mucosa (VM). We previously reported that in the comparison between skin and VM sensitization with 2,4‑dinitrofluorobenzene (DNFB), that preferentially induce Th1 responses, VM sensitization showed lower CHS including local ear swelling and T‐cell responses in the dLNs.^8^ Differential DC status may contribute to differences in CHS responses between skin and VM sensitization.

Several studies have been conducted on the DC features (e.g., distribution, phenotype, migration, and recruitment in the immune response) in oral and vaginal mucosae.^5^, ^9^, ^10^ Previous comparative study of fluorescein isothiocyanate (FITC) application between skin and oral mucosa showed that the number of CD11c^+^FITC^+^ migratory DCs from the oral mucosa was lower than that from the skin, while both oral mucosal and skin sensitization showed comparable results in phenotypes and kinetics of DCs on the dLNs.^9^ For VM, it has been reported that the number of LCs in the vaginal epithelium was lower than that in the skin epidermis in humans.^11^ In addition, a flow cytometric analysis of mice showed that the phenotypes, activation status, and the homeostatic renewal potential of DCs in steady state in the vaginal epithelium differed from those of DCs in the skin epidermis.^12^ However, little is known about the features of VM DCs in hapten‐induced contact allergy, despite being well researched in vaginal viral and bacterial infections^13^, ^14^ and the responses to protein antigen.^15^, ^16^

There are several immunosuppressive cytokines and cells assumed to be involved in the VM's sensitization ability to hapten. Transforming growth factor (TGF)‐β, an immunosuppressive cytokine, is known to contribute to the differentiation of DC precursors into macrophages, induction of M2 phenotype polarization, and inhibition of DC maturation and migration.^17^, ^18^, ^19^, ^20^, ^21^, ^22^ CD206 is a known marker for M2‐type macrophages, which have anti‐inflammatory and immunoregulatory functions and produce anti‐inflammatory cytokines such as interleukin‐10 and TGF‐β.^17^ These immunosuppressive cytokines and cells in hapten‐induced VM sensitization have not yet been investigated.

To understand the mechanism of VM sensitization in hapten‐induced contact allergy, we examined DC migration and recruitment after VM sensitization compared with skin sensitization. To investigate the immunosuppressive cytokines and cells after VM sensitization, we analyzed TGF‐β expression and CD206^+^ cell distribution in the VM.

## 2 MATERIALS AND METHODS

### 2.1 Animals

We purchased 6‐week‐old female C57BL/6J mice from Japan SLC and conducted the experiments when they were 7–10 weeks old. All experimental procedures were approved by the Kao Animal Care Committee, and all experiments followed the committee's guidelines.

### 2.2 Hapten‐induced sensitization

Skin sensitization was induced with 10 µl of 0.3% 2,4‑dinitrofluorobenzene (DNFB, Sigma‐Aldrich) in olive oil applied to shaved abdominal skin. To induce VM sensitization, we injected 10 µl of 0.3% DNFB into the vaginal cavity of the mice during the diestrus phase. To determine the estrous cycle phase, we collected vaginal smears with a swab and stained them with Giemsa stain solution (Fujifilm Wako Pure Chemical Corporation). We examined the stained cells and then determined the estrous cycle phase according to a previously established method.^23^ In the DC migration analysis, we sensitized the skin and VM with 10 µl of 1.0% FITC (DOJINDO) in acetone/dibutyl phthalate (1:1).

### 2.3 Flow cytometry

We collected inguinal and axillary LNs from the skin‐sensitized mice and iliac and inguinal LNs from the VM‐sensitized mice at 0, 6, 24, and 48 h after sensitization. To prepare single‐cell suspensions from the dLNs, the LNs were digested with collagenase type I (Sigma‐Aldrich, 400 U/L) at 37°C for 30 min and incubated with ethylenediaminetetraacetic acid (EDTA, final concentration 5 mM) for an additional 10 min.

**Figure 1:**
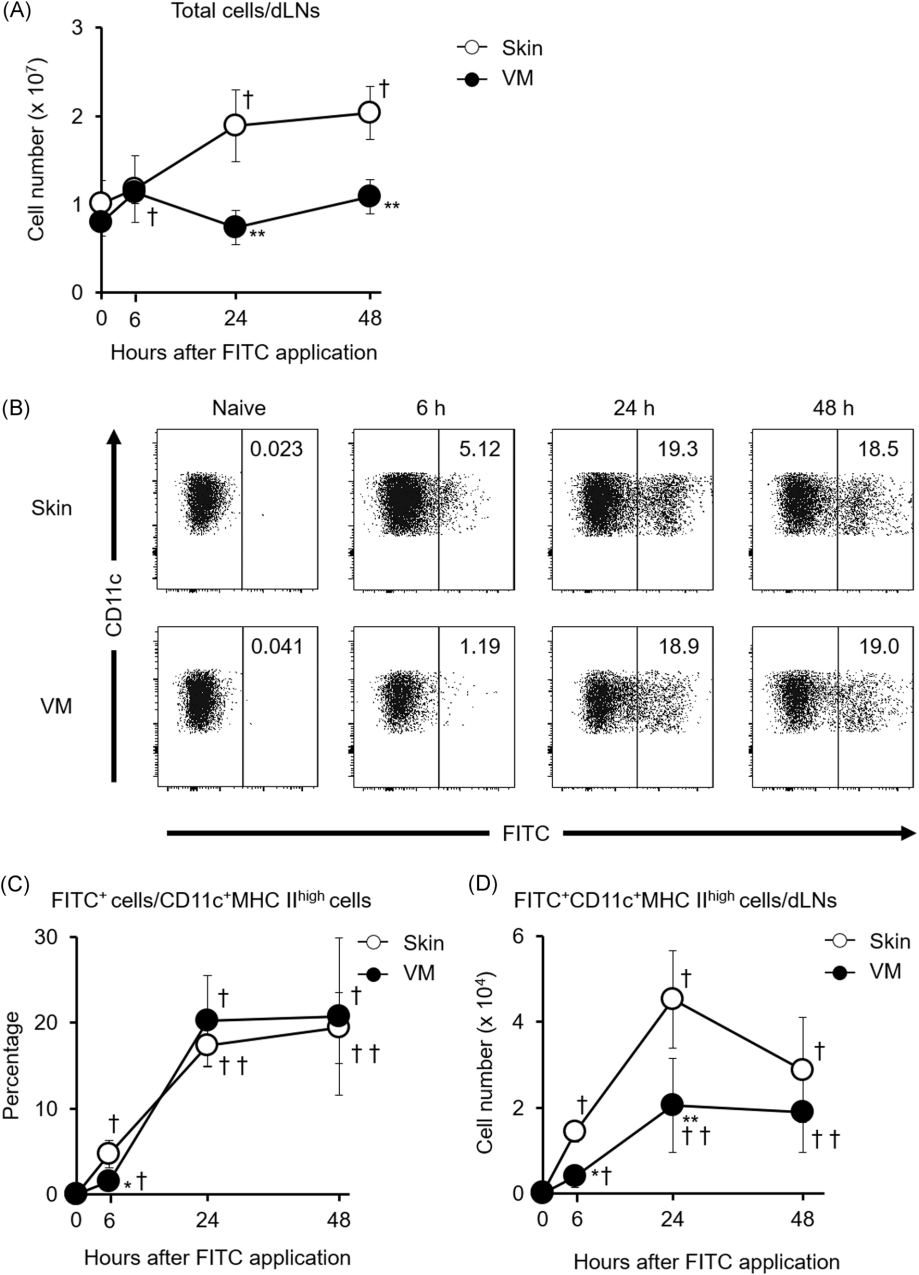
Changes in DC migration to dLNs after VM sensitization with FITC application compared with skin sensitization. (A) Changes in the number of total cells 6, 24, and 48 h after sensitization and without sensitization (0 h). (B) Representative flow cytometry data showing the percentage of FITC^+^ cells among 7‐ADD^−^MHC class II^high^CD11c^+^ cells in dLNs from naïve and sensitized mice (see Figure S1 for gating strategy). Changes in the proportion of FITC^+^ cells among 7‐ADD^−^MHC class II^high^CD11c^+^ cells (C) and the number of FITC^+^MHC class II^high^CD11c^+^ migratory DCs (D) 6, 24, 48 h after sensitization and without sensitization (0 h). Data show the mean values ± *SD* (*n* = 3–9). *p < .05, **p < .01 com*p*ared with skin‐sensitized mice. ^†^ *p* < .05, ^††^ *p* < .01 compared with 0 h (without sensitization)

Single cells were pretreated with an anti‐CD16/32 antibody (BD Biosciences) to block the Fc receptors and then stained with the following fluorochrome‐conjugated monoclonal antibodies purchased from BD Biosciences: anti‐CD11c (HL3)‐Pe‐Cy7 and MHC class II (M5/114.15.2)‐PE. Dead cells were labeled with 7‐amino‐actinomycin D (BD Biosciences). Stained samples were analyzed using a BD FACSVerse flow cytometer and FlowJo software (BD Biosciences). Figure 1 shows the gating strategy.

### 2.4 Immunohistochemistry

The shaved abdominal skin and VM were dissected from intact and DNFB‐sensitized mice that had been previously euthanized. The tissue samples were embedded in Tissue‐Tek O.C.T. Compound (Sakura Finetek Japan Co., Ltd.) and cryosectioned at a thickness of 7 µm. Cryostat sections were fixed in 4% paraformaldehyde in phosphate‐buffered saline (Fujifilm Wako Pure Chemical Corporation) for 3 min and treated with a blocking reagent, 1% bovine serum albumin (Fujifilm Wako Pure Chemical Corporation) in phosphate‐buffered saline. For the immunostaining with biotin‐conjugated anti‐MHC class II antibodies, we treated the sections with an avidin‐biotin blocking kit (Vector Laboratories). After blocking, the sections were incubated with primary antibodies at 4°C overnight, followed by appropriate streptavidin and secondary antibodies at room temperature for 30 min. We employed the following primary antibodies: biotin‐conjugated anti‐MHC class II (Thermo Fisher Scientific, M5/114.15.2), anti‐CD11c (BioLegend, N418), and anti‐CD206 (Abcam). We employed the following appropriate streptavidin and secondary antibodies: streptavidin‐RRX (Jackson ImmunoResearch Laboratories), Alexa Fluor 488‐conjugated anti‐Armenian hamster IgG (Abcam), and Cy5‐conjugated anti‐rabbit IgG (Jackson ImmunoResearch Laboratories). After incubation with the appropriate streptavidin and secondary antibodies, the sections were counterstained and mounted with VECTASHIELD Mounting Medium with DAPI (Vector Laboratories).

For the analysis of TGF‐β/CD206 double‐positive cells, we performed staining for TGF‐β with the Fc chimeric receptor comprising the ligand‐interacting ectodomains of TGF‐β type I (TβRI) and type II (TβRII) receptors fused with the Fc portion of immunoglobulin (TβRI‐TβRII‐Fc), which was kindly provided by Dr. Mikako Shirouzu (RIKEN) and traps all TGF‐β isoforms.^24^ After fixation and blocking, the sections were incubated with TβRI‐TβRII‐Fc at 4°C overnight, followed by Alexa Fluor 488‐conjugated anti‐human IgG (Jackson ImmunoResearch Laboratories) at room temperature for 30 min. After staining for TGF‐β, the sections were incubated with the anti‐CD206 antibody at room temperature for 1 h, followed by the Cy5‐conjugated anti‐rabbit IgG antibody at room temperature for 30 min.

Fluorescence images were acquired on an LSM 880 confocal microscope (Carl Zeiss Co., Ltd.). All fluorescence images were optimized and analyzed using ImageJ/Fiji (NIH) software. For the quantitative analysis, we counted MHC class II^+^ cells in the epidermis and epithelium as LCs and those in the dermis and lamina propria as dermal DCs and submucosal DCs, respectively. We also counted the CD206^+^ cells in the basal portion of the lamina propria (approximately 150–300 μm from the basement membrane). The results represent the number of cells per unit length of the basement membrane.

### 2.5 Quantitative real‐time PCR

We extracted total RNA from the shaved abdominal skin and VM of the intact and DNFB‐sensitized mice 6 and 12 h after sensitization with the RNeasy Fibrous Tissue Mini Kit (Qiagen). We prepared cDNA from total RNA samples using a High‐Capacity cDNA Reverse Transcription Kit (Applied Biosystems). We performed a quantitative real‐time PCR using a Taqman Gene Expression Assay and the QuantStudio 5 Real‐Time PCR system (Applied Biosystems), employing the following TaqMan probes: TGF‐β1 (Mm01178820_m1), TGF‐β2 (Mm00436955_m1), TGF‐β3 (Mm00436960_m1), and β‐actin (Mm00607939_s1). We employed β‐actin as an endogenous control. The results represent normalized mRNA expression relative to the skin‐intact groups by the $2^{‐{\mathrm{\Delta \Delta C}}_{t}}$method.

### 2.6 Statistics analysis

The statistical analyses were performed using the Mann–Whitney *U*‐test. A *p *value < .05 was considered statistically significant.

## 3 RESULTS

### 3.1 Dendritic cell migration to draining lymph nodes after skin and vaginal mucosa sensitization

We analyzed DC migration after skin and VM sensitization using FITC, which is typically employed to identify migratory DCs within dLNs in sensitization.^25^ After applying FITC to the skin, the number of total cells in the dLNs increased for 48 h; however, the continuous increase was not observed after VM sensitization and the number of cells was significantly lower in the VM‐sensitized mice than in the skin‐sensitized mice at 24 and 48 h (Figure 1A). The number of MHC class II^high^CD11c^+^ DCs in dLNs increased and peaked at 24 h after skin sensitization, while the significant increase was not observed after VM sensitization (data not shown). Both skin and VM sensitization increased the proportions of FITC^+^ cells among MHC class II^high^CD11c^+^ cells in dLNs (Figure 1B,C). The proportions of FITC^+^ cells at 24 and 48 h were comparable between the skin‐sensitized and VM‐sensitized mice, while the proportion at 6 h was lower in the VM‐sensitized mice. The number of MHC class II^high^CD11c^+^FITC^+^ migratory DCs in the dLNs also increased after both skin and VM sensitization, and the increased levels achieved peaks at 24 h (Figure 1D). However, the number of MHC class II^high^CD11c^+^FITC^+^ migratory DCs in the dLNs was lower in the VM‐sensitized mice than in the skin‐sensitized mice at 6 and 24 h, while there was no significant change at 48 h after sensitization between the skin and VM. These data indicate that DC migration was limited in VM sensitization.

### 3.2 Distribution and number of dendritic cells in the intact and sensitized skin and vaginal mucosa

**Figure 2:**
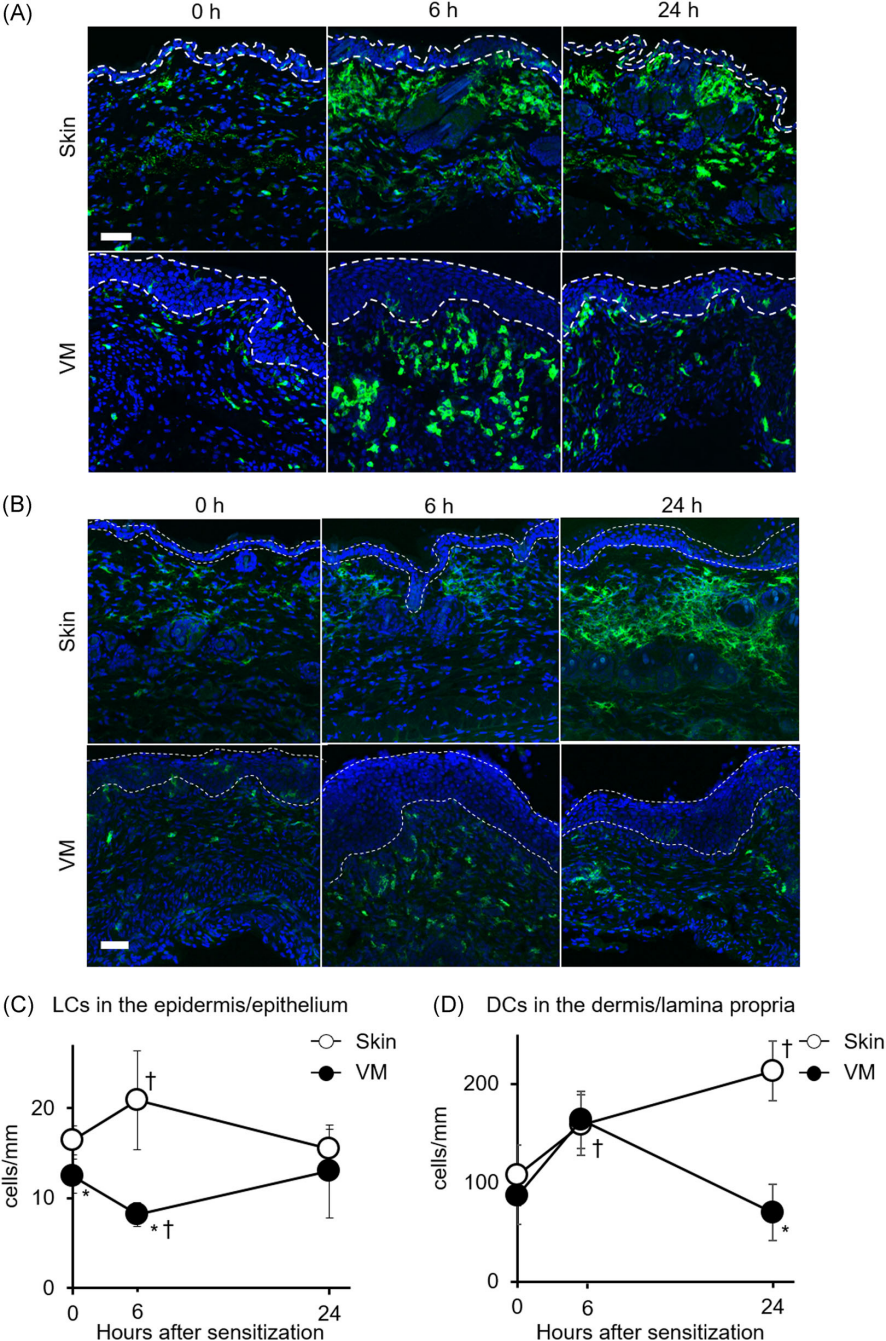
Changes in DC distribution and number in the skin and VM after DNFB sensitization. Representative images of DCs immunostained using anti‐MHC class II (green) (A) and anti‐CD11c (green) (B), and counterstained with DAPI (blue) in sections of intact and DNFB applied tissues at 6 and 24 h. The dotted areas indicate the epidermis and epithelium. Scale bar, 50 µm. (C) Quantification of the number of MHC class II^+^ LCs per unit length of basement membrane in the epidermis and epithelium. (D) Quantification of the number of MHC class II^+^ dermal and submucosal DCs per unit length of basement membrane in the dermis and lamina propria. Data show the mean values ± *SD* (*n* = 3). *p < .05 compared with skin‐sensitized mice. ^†^ *p* < .05 com*p*ared with 0 h (intact skin/VM)

To assess the distribution and number of DCs in the VM compared with the skin at steady state and after sensitization, we analyzed the DCs in the skin and VM by immunohistochemistry with an antibody to MHC class II. MHC class II^+^ cells were located within both the epidermis/epithelium and dermis/lamina propria (Figure 2A). After sensitization, we observed MHC class II^+^ DC recruitment at 6 and 24 h in the skin dermis and at 6 h in the vaginal lamina propria. CD11c immunostaining showed similar results (Figure 2B). The quantitative analysis showed that the number of MHC class II^+^ LCs in the vaginal epithelium was significantly lower than that in the skin epidermis at steady state (0 h) and at 6 h after sensitization (Figure 2C). For the dermal DCs in the skin dermis, the number of MHC class II^+^ dermal DCs continually increased until 24 h after DNFB application to the skin, and the number at 24 h was 2.0‐fold higher than at steady state. After VM sensitization, the number of MHC class II^+^ submucosal DCs in the lamina propria increased dramatically by 1.9‐fold at 6 h compared with the intact VM (0 h) and decreased at 24 h to a similar level to that of the intact VM (Figure 2D). These findings indicate that continuous recruitment of DCs was limited in VM sensitization.

### 3.3 TGF‐β expression and CD206‐expressed cells after sensitization

**Figure 3:**
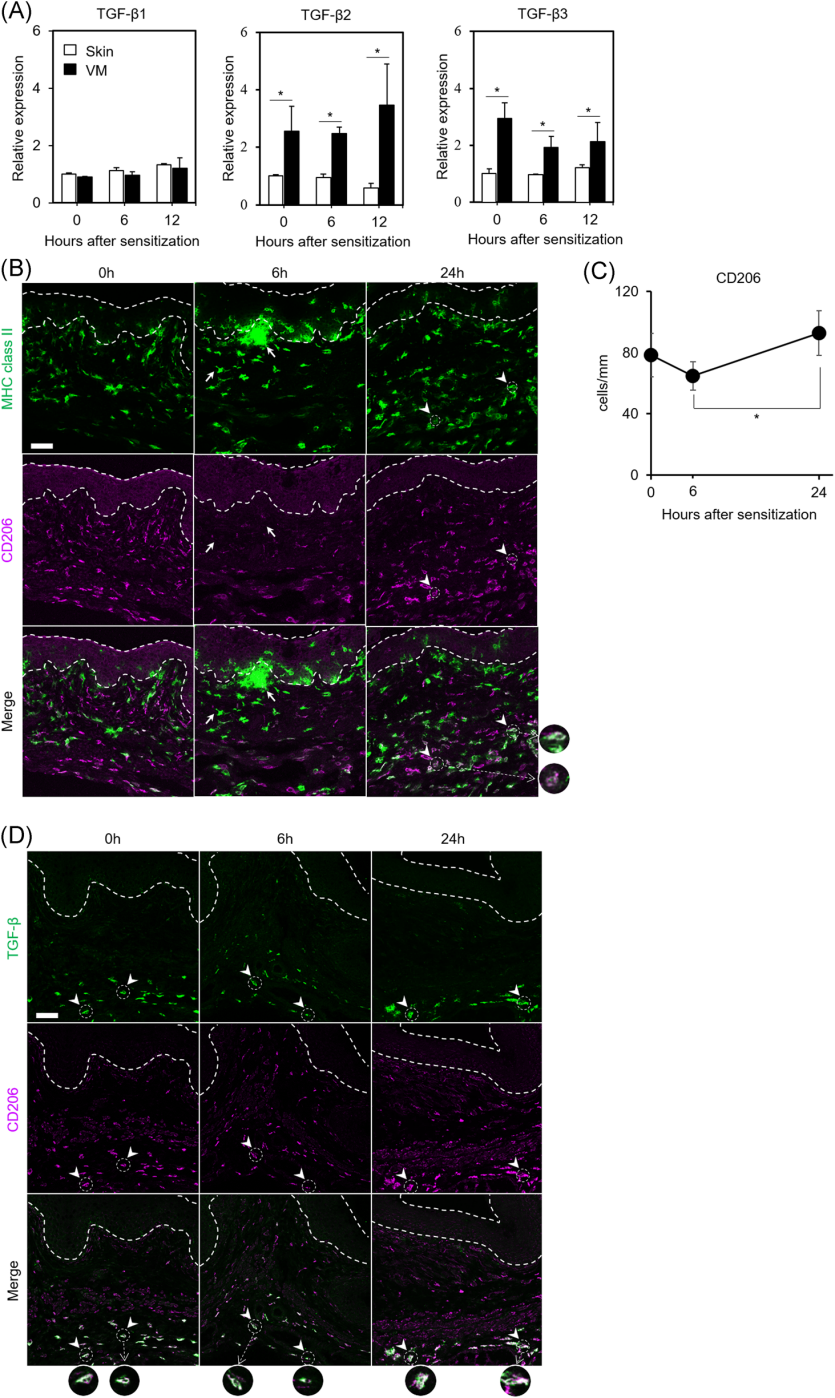
Changes in TGF‐β expression and CD206‐expressed cell distribution after sensitization. (A) mRNA expression levels of TGF‐β in the skin and VM at the indicated time points after sensitization. mRNA levels of TGF‐β1, ‐β2, and ‐β3 in the skin and VM by quantitative real‐time PCR are shown as relative expression to intact skin (0 h). Data show the mean values ± *SD* (*n* = 3). *p < .05. (B) MHC class II and CD206 expression in before (0 h) and 6 and 24 h after VM sensitization. Representative images of cells immunostained using anti‐MHC class II (green) and anti‐CD206 (magenta) are shown. Arrows indicate representative MHC class II^+^CD206^−^ cells. Arrowheads indicate representative MHC class II^+^CD206^+^ round‐type cells. The dotted areas indicate the epithelium. Scale bar; 50 μm (C) Quantification of the number of CD206^+^ cells per unit length of basement membrane in the basal portion of the lamina propria. Data show the mean values ± *SD* (*n* = 3). *p < .05. (D) TGF‐β and CD206 expression before (0 h) and 6 and 24 h after VM sensitization. Representative images of cells stained with TβRI‐TβRII‐Fc (green) and anti‐CD206 (magenta) are shown. Arrowheads indicate representative TGF‐β/CD206 double‐positive cells. The dotted areas indicate the epithelium. Scale bar; 50 μm

To investigate the reason for limited number of VM DCs, we first examined TGF‐β expression, which is known to inhibit DC functions, and observed that the VM expressed 1.9‐ to 3.5‐fold higher levels of TGF‐β2 and TGF‐β3 than the skin at all analyzed time points (Figure 3A). TGF‐β contributes to the differentiation of DC precursors into macrophages and the induction of M2 phenotype polarization.^17^, ^18^, ^20^ We therefore expected the existence of CD206^+^ cells are involved in limiting supply of VM DCs, and analyzed CD206 and MHC class II co‐expression in the VM after sensitization by immunohistochemistry. MHC class II^+^CD206^‐^ cells, which are expected to respond to hapten, were mainly observed in the apical portion of the lamina propria (beneath the basement membrane) especially at 6 h after VM sensitization (Figure 3B). Moreover, we observed MHC class II^+^CD206^+^ cells in the basal portion of the lamina propria, especially at 24 h after VM sensitization, indicating that local recruitment of CD206^+^ cells had occurred during VM sensitization. The quantitative analysis also showed that the number of CD206^+^ cells in the basal portion of the lamina propria at 24 h was significantly higher than that at 6 h after VM sensitization (Figure 3C). CD206^+^ macrophages, induced by TGF‐β, are also an important producer of TGF‐β.^18^ Thus, to characterize TGF‐β‐expressing cells in the VM, we performed double‐label fluorescent immunohistochemistry using an anti‐CD206 antibody and TβRI‐TβRII‐Fc, which traps all TGF‐β isoforms.^24^ Most of the cells positive for TβRI‐TβRII‐Fc were CD206 positive at all analyzed time points, indicating that CD206^+^ cells produce TGF‐β in the VM (Figure 3D). These results suggest that TGF‐β‐producing CD206^+^ cells were involved in limiting DC migration and recruitment after VM sensitization.

## 4 DISCUSSION

The present study showed differences in the features of DCs between skin and VM sensitization with haptens. The flow cytometry revealed a lower number of MHC class II^high^CD11c^+^FITC^+^ migratory DCs, while the histological analysis showed that MHC class II^+^ submucosal DCs rapidly decreased after VM sensitization compared with skin sensitization. Moreover, we observed high TGF‐β expression levels, increment of CD206^+^ cells, and TGF‐β expression in CD206^+^ cells in the VM during sensitization. These results indicate that VM sensitization limits DC migration and recruitment compared with skin sensitization and that TGF‐β‐producing CD206^+^ cells might contribute to the suppression.

We previously reported that VM sensitization induced less ear swelling and fewer proliferating cells in dLNs than those in skin sensitization using a DNFB‐induced CHS model.^8^ Consistent with the previous report, DC migration and recruitment after sensitization were limited in the VM compared with the skin (Figures 1 and 2). These observations indicated that sensitization ability in the VM might be lower than in the skin based on mechanism. This is the first report regarding the DC response to haptens in the VM sensitization phase of CHS.

Studies on the oral (buccal and sublingual) mucosal application of FITC in mice reported that the number of MHC class II^+^ DCs in the submucosal layer increased, peaking 6 h after the FITC painting and decreased at 24 h.^9^, ^26^ Moreover, the number of FITC^+^CD11c^+^ DCs in dLNs was lower in buccal mucosal sensitization than in ear skin sensitization.^9^ In the present study, we reported that VM sensitization also showed similar results to oral mucosal sensitization. Both vaginal and oral mucosae belong to type II mucosae covered by a stratified squamous epithelium, which share many features with the skin, and differ from type I mucosae, such as the intestinal and respiratory mucosae, which are covered by simple epithelium.^27^ Therefore, these observations suggest that type II mucosae might have a common mechanism in DC recruitment and migration after hapten sensitization.

It is still unclear why continuous DC migration and recruitment were limited after VM sensitization compared with skin sensitization. It had been reported that TGF‐β contributes to the inhibition of DC functions, including migration, maturation, and antigen presentation.^19^, ^21^, ^22^ TGF‐β isoforms (β1, β2, and β3) are expressed in the VM^28^ and inhibit vaginal antigen‐presenting cells in ovalbumin‐specific T‐cell proliferation.^29^ In repeated antigen painting on the sublingual mucosa, CD206^+^ tolerogenic macrophage cells were induced, and CD206^+^ cells suppressed DC function.^30^ In this study, we found that the expression levels of TGF‐β2 and β3 were higher than those of the skin and that CD206^+^ cells expressed TGF‐β in the VM (Figure 3A,D). We also found the increment of MHC class II^+^CD206^+^ cells in the basal portion of the lamina propria at 24 h after VM sensitization (Figure 3B,C). These results suggest that CD206^+^ cells suppress DC migration and recruitment via TGF‐β2 and TGF‐β3 in the VM after sensitization.

Along with protecting against infection and other noxious environmental insults, VM is known to be an inductive site for tolerance, and the effect is hormonally regulated.^31^ TGF‐β‐producing CD206^+^ cells can contribute to tolerance induction in the VM, which is controlled by the hormonal cycle. The mouse estrous cycle is divided into four stages: proestrus, estrus, metestrus, and diestrus.^23^ Diethylstilbestrol, a synthetic estrogen, increases the expression of TGF‐β1, ‐β2, and ‐β3,^23^ and estrogen levels peak during the proestrus stage.^32^ Estradiol inhibits antigen presentation in the VM, which is mediated through the local production of TGF‐β by vaginal cells.^24^ Therefore, it is expected that the induction of TGF‐β‐producing CD206^+^ cells would change during the estrous cycle depending on estrogen levels, although we used mice in the diestrus phase in this study. Further investigations will be required to reveal the effect of the estrous cycle on hapten sensitization and tolerance induction in the VM.

In summary, we have examined the features of DCs in VM sensitization compared with skin sensitization and found that the number of migratory DCs was reduced and that submucosal DCs were rapidly decreased after VM sensitization. In addition, high levels of TGF‐β expression, MHC class II^+^CD206^+^ cells, and TGF‐β/CD206 double‐positive cells were observed after sensitization in the VM. Our findings suggest that sensitization ability in the VM might be lower than in the skin through the prevention of continuous DC migration and recruitment and that TGF‐β‐producing CD206^+^ cells might contribute to the suppression of DC migration and recruitment after VM sensitization. These findings could improve our understanding of the VM sensitization mechanism in contact allergy and tolerance induction in the VM.

## CONFLICTS OF INTEREST

The authors declare no conflicts of interest.

## AUTHOR CONTRIBUTIONS

*Study conception and Experimental design*: Kanako Nakayama, Taku Nishijo, Masaaki Miyazawa, and Miyuki Azuma. *Acquisition and analysis of data*: Kanako Nakayama and Taku Nishijo. *Interpretation of data*: All authors. *Writing*: Kanako Nakayama.

## Supporting information

Supplementary information.

## Data Availability

The data that support the findings of this study are available from the corresponding author upon reasonable request.
