# Supplementary figures and images for: Hapten sensitization to vaginal mucosa induces less recruitment of dendritic cells accompanying TGF‐β‐expressing CD206+ cells compared with skin
Source: Immun Inflamm Dis. 2022 Mar 10;10(4):e605. doi: 10.1002/iid3.605 (PMC8959427; doi:10.1002/iid3.605)

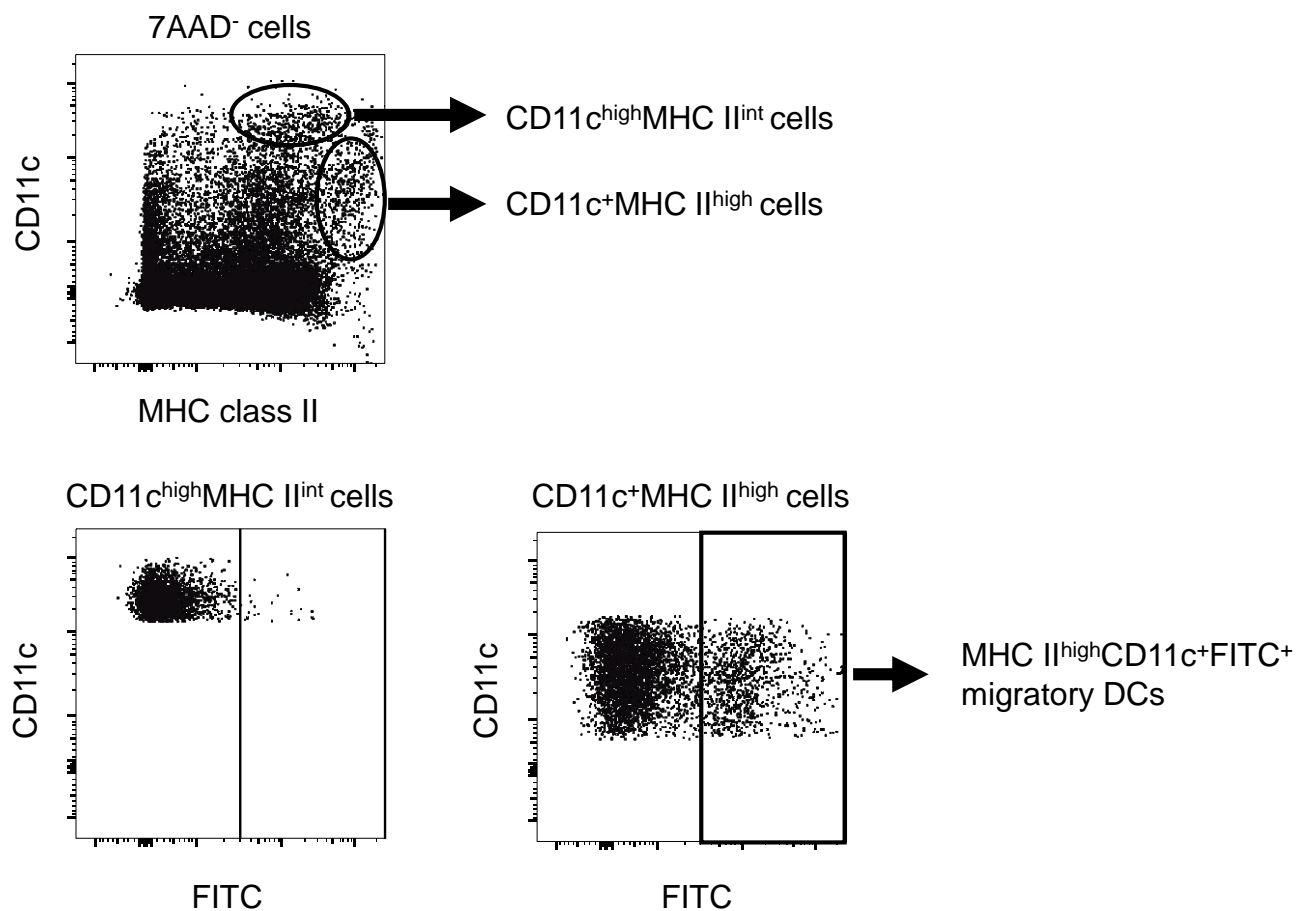

**Figure 1.** Gating strategy for identification of migratory DCs in the dLNs.

Supplement: Supplementary file 1 — Supplementary information. [file IID3-10-e605-s001.pdf]
